# Natural outer membrane permeabilizers boost antibiotic action against irradiated resistant bacteria

**DOI:** 10.1186/s12929-019-0561-6

**Published:** 2019-09-09

**Authors:** Hala A. Farrag, Nagwa Abdallah, Mona M. K. Shehata, Ebthag M. Awad

**Affiliations:** 1 Drug Radiation Research Department, National Center for Radiation Research and Technology, Atomic Energy Authority, P.O. Box 29, Nasr City, Cairo, Egypt; 2 Microbiology Department, Faculty of Science, Ain Shams University, Cairo, Egypt

**Keywords:** Pathogenic gram-negative bacilli, Outer membrane permeability, Beta lactam resistance, Permeabilizers, Natural beta lactamase inhibitors, In vitro gamma irradiation

## Abstract

****Background**:**

This study sought to develop new strategies for reverting the resistance of pathogenic Gram-negative bacilli by a combination of conventional antibiotics, potent permeabilizers and natural beta lactamase inhibitors enhancing the activity of various antibiotics.

**Methods:**

The antibiotic susceptibility in the presence of natural non-antibacterial tested concentrations of phytochemicals (permeabilizers and natural beta lactamase inhibitors) was performed by disk diffusion and susceptibility assays. Thymol and gallic acid were the most potent permeabilizers and facilitated the passage of the antibiotics through the outer membrane, as evidenced by their ability to cause LPS release, sensitize bacteria to SDS and Triton X-100.

**Results:**

The combination of permeabilizers and natural beta lactamase inhibitors (quercetin and epigallocatechin gallate) with antibiotics induced greater susceptibility of resistant isolates compared to antibiotic treatment with beta lactamase inhibitors alone. Pronounced effects were detected with 24.4 Gy in vitro gamma irradiation on permeability barrier, beta lactamase activity, and outer membrane protein profiles of the tested isolates.

**Conclusions:**

The synergistic effects of the studied natural phytochemicals and antibiotics leads to new clinical choices via outer membrane destabilization (permeabilizers) and/or inactivation of the beta lactamase enzyme, which enables the use of older, more cost-effective antibiotics against resistant strains.

## Background

The development and spread of antibiotic-resistant bacteria are pressing public health problems worldwide. Gram-negative bacteria are important pathogenic bacteria with a unique outer membrane (OM) that makes them inherently resistant to many antimicrobial agents. Hydrophilic antibacterial agents are prevented from entering through the outer membrane by the lipopolysaccharide layer (LPS) and the underlying phospholipids, whereas hydrophobic agents are excluded by outer membrane proteins [1]. LPS, also termed endotoxins, are the main components of the outer leaflet in the OM of Gram-negative bacteria. LPS form a permeation barrier and play an essential role in drug resistance [2]. To improve the efficacy of antibiotics, it is necessary to explore methods that improve the diffusion of antibiotics and bypass the bacterial membrane barrier, which is responsible for the general antibiotic resistance of Gram-negative bacteria. Permeabilizers are compounds that weaken the OM and can nonspecifically enhance the permeability of bacterial cells to exogenous products, including antimicrobial agents. They may therefore potentiate the antibacterial activity of antibiotics that interact with intracellular targets mainly due to the perturbation of the lipid fraction of the cell membrane as they disintegrate the LPS layer. Additionally, owing to their lipophilic character, they can increase membrane permeability. The use of OM permeabilizers, in combination with antibiotics, may provide additional means of controlling growth of Gram-negative bacteria [3–6].

All β-lactam antibiotics interfere with bacterial cell wall synthesis by inhibiting a number of bacterial enzymes, known as transpeptidases, that are essential for the synthesis of peptidoglycan in the bacterial cell wall; this leads to dissolution of the cell wall and bacterial lysis. β-lactam antibiotics are different in their spectrum of activity, their effects on Gram-negative rods and susceptibility to bacterial β-lactamase enzymes. β-lactamases are the most common cause of bacterial resistance to β-lactam antimicrobial agents [7]. β-lactamases interact with β-lactam antibiotics in reactions that result in the hydrolysis of the antibiotic to form an inactive chemical substance no longer possessing antibacterial activity [8]. Extended-spectrum β-lactamases (ESBLs) are able to hydrolyze a broader spectrum of β-lactam antibiotics than the simple parent β-lactamases from which they are derived. ESBL genes are localized on plasmids, and this route of transmission has enabled the extremely rapid spreading of resistant pathogens all over the world. A successful strategy for combating β-lactamase-mediated resistance is the use of agents designed to bind at the active site (the β-lactams ring); these agents are called beta lactamase inhibitors (tazobactam, clavulanic acid and sulbactam). The function of these enzyme inhibitors is the inactivation of the beta lactamase in the periplasmic space. Thus, they can serve to protect the familiar beta lactam antibiotics from hydrolysis by penicillinases or broad-spectrum beta lactamases. However, bacterial resistance to these suicide inhibitors has significantly increased in recent years. This could be related to their frequent use. Furthermore, the appearance of ESBL increase resistance against various bacterial infections. Therefore, there have been considerable efforts to discover other inhibitors of β-lactamases to prevent inactivation of β-lactams by β-lactamases [3, 9]. To this end, the aim of this study was to improve the efficacy of antibiotics and decrease bacterial resistance through studying the role of natural non-antibacterial permeabilizers in releasing the lipopolysaccharide and increasing the outer membrane permeability of gamma-irradiated and non-irradiated Gram-negative bacterial isolates. We further analyzed the impact of natural beta lactamase inhibitors to potentiate the activity of antibiotics to which resistance has developed.

## Methods

### Isolation and antibacterial susceptibility test of pathogenic gram-negative bacilli

Bacterial isolation from different sample sources and sensitivity test using the Kirby-Bauer method (Disk diffusion test) against 23 antibiotics representing several modes of action were carried out as mentioned in [4].

### Detection of extended-spectrum beta lactamase (ESBL) activity by different methods

ESBL production was examined by Double disk synergy tests (DDST) [10], Modified double disk synergy tests (MDDT) [11], Phenotypic confirmatory tests (combined disk method) [12], and the Nitrocef disks method [13]. Fifteen identified selected bacterial isolates from previous study [4] were used for further experiments in this study and representing different species; [*Escherichia coli* (3), *Acinetobacter baumannii* (3), *Pseudomonas fluorescens* (1), *Pseudomonas aeruginosa* (4), *Klebsiella pneumoniae* (1), *Enterobacter sakazakii* (1), and *Enterobacter cloacae* (2)] to distinguish between beta lactamase producer and non-producer isolates.

### Examination of antibacterial activity of certain permeabilizers and natural beta lactamase inhibitors (phytochemicals)

Antibacterial activity of the permeabilizers [gallic acid (500, 600, 700 μg/mL), ellagic acid (30, 40, 50 μM), thymol (400, 500, 600 μg/mL), chitosan (50, 100, 250 ppm), Ethylenediaminetetraacetic acid (EDTA) (0.1, 0.5, 1 mM) and sorbic acid (2, 5, 10 mM)], and natural beta lactamase inhibitors [quercetin (25, 50, 100 μg/mL), and epigallocatechin gallate (25, 50,100 μg/mL) were determined (50 μL each) against the studied bacterial isolates, as described by [14], using both disk diffusion (6 mm filter paper discs) and agar well diffusion (10 mm diameter wells) in nutrient agar (NA) plates.

### Reduction of antimicrobial resistance

#### Evaluation of synergistic interactions between non-antibacterial permeabilizers, natural beta lactamase inhibitors and different antibiotics

a: *Non-beta lactamase producing isolates (antibacterial permeability and antibiotics*).

Ten identified multidrug resistant pathogenic isolates were subjected to increasing levels of several non–antibacterial permeabilizers [Chitosan 100 ppm, (EDTA) 0.1 mM, Ellagic acid 40 μM, Gallic acid 600 μg/mL, Sorbic acid 5 mM, Thymol 500 μg/mL] to assess their susceptibility to different selected antibiotics with different modes of action as described by [15]. The selected concentrations had no baseline antibacterial activity.

b: *Beta lactamase producing isolates.*

Five multidrug resistant isolates were subjected to testing of the effects of natural beta lactamase inhibitors (epigallocatechin gallate 50 μg/mL and quercetin 50 μg/mL) alone and in combination with permeabilizers (gallic acid 600 μg/mL and thymol 500 μg/mL) to reduce their bacterial cell wall resistance to beta lactam and standard beta lactamase inhibitor antibiotic discs [piperacillin (PRL) (100 μg), piperacillin/ tazobactam (TZP) (100/10 μg), cefoperazone (CFP) (75 μg), cefoperazone/sulbactam (SCF) (75 /30 μg)] as described by [16].

#### Irradiation source

A cesium 137 (^137^Cs) Gamma cell 40, Atomic Energy of Canada Limited, commercial product located at the National Center for Radiation Research and Technology (Nasr City, Cairo, Egypt) was used for irradiation of several identified Gram-negative bacterial isolates.

#### Effect of in vitro gamma irradiation on the studied multidrug-resistant isolates

By using the linear quadratic (LQ) formula described by [17], an in vitro low radiation dose equivalent to 24.4 Gy, which is biologically equivalent to the 70 Gy in vivo fractionated multiple therapeutic dose used in the cancer treatment protocol of certain cancer patients, was used for irradiation. This source was used at a dose rate of 0.75 rad/sec for the experiments.

Bacterial cultures were divided into two groups, one before irradiation (control group) and the other after irradiation, and each test was performed twice before and after irradiation.

#### Effect of gamma irradiation on the production of ESBLs

The five studied multidrug resistant producing isolates were assessed for their production of ESBLs by DDST and MDDT after in vitro gamma irradiation.

#### Determination of minimum inhibitory concentrations (MICs) for selected antibiotics

Four non-beta lactamase producers [*Escherichia coli*_3_, *Acinetobacter baumannii*_5_, *Pseudomonas aeruginosa*_10_, and *Enterobacter sakazakii*_14_] and three producer bacterial isolates [*Escherichia coli*_2_, *Pseudomonas aeruginosa*_8_, and *Enterobacter cloacae*_13_] were investigated in this experiment using antibiotics that were determined in previous work [4] to be highly resistant for these organisms [18]. MICs break points were determined according to [19].

#### Evaluation of MICs of selected antibiotics in the presence of non-antibacterial permeabilizers and natural beta lactamase inhibitors for certain bacterial isolates

The activity of gallic acid and thymol as a modulator of antibiotic resistance against four selected irradiated and non-irradiated non-beta lactamase producing bacterial isolates, as described by [20], were determined in combination with cefotaxime (CTX, Avants, Cairo, Egypt), cefoperazone (CFP, Pfizer, Cairo, Egypt) and erythromycin (E, National Organization for Drug Control and Research [NODCAR], Cairo, Egypt) by the agar diffusion method. Additionally, the MICs of cefoperazone (CFP) and piperacillin (PRL, National Organization for Drug Control and Research [NODCAR], Cairo, Egypt) in the presence of natural beta lactamase inhibitors (epigallocatechin gallate and quercetin) alone and in combination with selected permeabilizers (gallic acid and thymol) were assessed for three selected beta lactamase producers before and after irradiation.

#### Permeability assay of the outer membrane in the presence of lytic agents of non-beta lactamase producers treated with gallic acid and thymol

Two multidrug resistant non-beta lactamase producing bacterial isolates were subjected to permeability assays. This method was utilized to determine the permeability properties of the outer membrane in the presence of thymol and gallic acid via their increased susceptibility to the bacteriolytic action of detergents [Triton X-100 & sodium dodecyl sulphate (SDS)] and to the cell wall-degrading action of lysozyme. All bacteriolytic agents were purchased from Sigma-Aldrich, St. Louis, USA. The bacteriolytic effect was assayed on Nunclon microtiter plates (Nunc) by measuring the OD630 of bacterial cultures as previously described [21]. Cell death caused by the sudden influx of these lytic agents was determined by measuring the decrease in optical density (OD) (Relative turbidity %).

#### Release of lipopolysaccharide

The potential release of OM components (e.g., LPS) was investigated by silver staining of cell-free supernatants after treatment with radiation or permeabilizers [thymol (500 μg/mL), gallic acid (600 μg/mL), EDTA (0.1 mM) and chitosan (100 ppm)]. The samples were analyzed by SDS-PAGE via precast 12% acrylamide gels. Ten μL of lysate was applied to the gel. The gel with proteinase K-treated samples was stained with silver staining [22].

#### Beta lactamase assays using the peroxidase-chromophore decolorization method of irradiated and non-irradiated beta lactamase producers

This testing was carried out on two multidrug-resistant isolates producing beta lactamase enzyme before and after 24.4 Gy in vitro gamma irradiation in the presence of a permeabilizer (thymol) and a beta lactamase inhibitor (quercetin) for qualitative and quantitative analysis of the production of the open beta lactam ring end product resulting from the hydrolysis of a beta lactam antibiotic, as described by [23].

### Statistical analysis

Statistical analysis was performed via computer using the Paired t test, Chi-squared test and One-way analysis of variance (One-way ANOVA). The means and standard deviations (SD) were calculated using SAS software version 9 (SAS Institute, Cary, N.C., USA).

## Results

Data representing the frequency of the identification of antibiotic-resistant and -susceptible bacterial isolates against different antibiotics by mode of action were carried out and shown in previous work [4].

In this study, extended-spectrum β-lactamases (ESBLs) production by 15 selected bacterial isolates representing five species is shown in Table 1.

**Table 1:** Screening for extended-spectrum β-lactamases (ESBLs) production by certain pathogenic bacterial isolates using different methods

Isolates no.	Bacterial Species (GNB)	DDST result	MDDT result	Combined disc method		Nitrocefin test result	Production of beta- lactamase
				CFP-SCF	PRL-TZP		
1	*Escherichia coli*	–	–	–	–	–	non-producer
2	*Escherichia coli*	+	+	+	+	+	Producer
3	*Escherichia coli*	–	–	–	–	–	non-producer
4	*Acinetobacter baumannii*	+	+	+	+	+	Producer
5	*Acinetobacter baumannii*	–	–	–	–	–	non-producer
6	*Acinetobacter baumannii*	–	–	–	–	–	non-producer
7	*Pseudomonas* spp.	–	–	–	–	–	non-producer
8	*Pseudomonas* spp.	+	+	+	+	+	Producer
9	*Pseudomonas* spp*.*	+	+	+	+	+	Producer
10	*Pseudomonas* spp.	–	–	–	–	–	non-producer
11	*Pseudomonas* spp.	–	–	–	–	–	non-producer
12	*Klebsiella pneumoniae*	–	–	–	–	–	non-producer
13	*Enterobacter* spp.	+	+	+	+	+	Producer
14	*Enterobacter* spp.	–	–	–	–	–	non-producer
15	*Enterobacter* spp.	–	–	–	–	–	non-producer

*DDST* Double disk synergy test, *MDDT* Modified double disk synergy test, *CFP* Cefoperazone, *SCF* Cefoperazone sulbactum. *PRL* Piperacillin, *TZP* Piperacillin tazobactam, *GNB* Gram- negative bacilli

Sub-MICs for permeabilizers were determined for the five selected species to study their effect on uptake of antibiotics by bacterial outer membrane (Table 2). These concentrations [gallic acid (≤ 600 μg/mL), ellagic acid (≤ 40 μM), thymol (≤ 500 μg/mL), chitosan (≤ 100 ppm), EDTA (≤ 0.1 mM), and sorbic acid (≤ 5 mM)] were selected for further studies to guarantee that any inhibitory effect and antibacterial activity would be due to the antibiotic action alone and ensure that the permeabilizers had no inherent antibacterial activity.

**Table 2:** Examination of antibacterial activity of some selected permeabilizers (phytochemicals) through agar diffusion method against certain pathogenic Gram-negative bacilli

Permeabilizers conc.	Microorganisms														
	*Escherichia coli*			*Acinetobacter baumannii*			*Pseudomonas* spp.					*Klebsiella pneumoniae*	*Enterobacter* spp.		
	1	2	3	4	5	6	7	8	9	10	11	12	13	14	15
Gallic acid															
500 μg/ml	–	–	–	–	–	–	–	–	–	–	–	–	–	–	–
600 μg/ml	–	–	–	–	–	–	–	–	–	–	–	–	–	–	–
700 μg/ml	–	–	–	–	–	–	–	–	–	–	–	–	–	–	+
Ellagic acid															
30 μM	–	–	–	–	–	–	–	–	–	–	–	–	–	–	–
40 μM	–	–	–	–	–	–	–	–	–	–	–	–	–	–	–
50 μM	–	–	–	–	–	–	–	–	–	–	–	–	–	–	+
Thymol															
400 μg/ml	–	–	–	–	–	–	–	–	–	–	–	–	–	–	–
500 μg/ml	–	–	–	–	–	–	–	–	–	–	–	–	–	–	–
600 μg/ml	–	–	–	–	–	–	–	–	–	–	–	+	–	–	+
Chitosan															
50 ppm	–	–	–	–	–	–	–	–	–	–	–	–	–	–	–
100 ppm	–	–	–	–	–	–	–	–	–	–	–	–	–	–	–
250 ppm	–	–	–	–	–	+	–	+	–	–	–	–	–	–	–
EDTA															
0.1 mM	–	–	–	–	–	–	–	–	–	–	–	–	–	–	–
0.5 mM	–	–	–	–	–	–	–	–	–	–	–	–	–	–	–
1 mM	–	–	–	–	–	–	–	–	–	–	–	–	–	–	–
Sorbic acid															
2 mM	–	–	–	–	–	–	–	–	–	–	–	–	–	–	–
5 mM	–	–	–	–	–	–	–	–	–	–	–	–	–	–	–
10 Mm	–	+	–	–	–	–	–	–	–	–	–	+	–	–	+

(+) susceptibility (in case of disc method inhibition zone > 6 and in case of agar method inhibition zone > 10)

(−) absence of susceptibility

Additionally, the effects of quercetin (50 μg/mL) and epigallocatechin gallate (50 μg/mL), as natural β-lactamase inhibitors, on ESBL-producing isolates were assessed; these phytochemicals did not show any antibacterial activity. The results obtained in the current study revealed that, for the 10 non-beta lactamase producing isolates, gallic acid and thymol were the most potent permeabilizers—they potentiated the activity of all tested antibiotics against multi-drug resistant (MDR) (100% resistant) bacterial pathogens with changes towards susceptibility. The max. Mean inhibition zone (mm) for antibiotics alone vs. the synergistic effect of gallic acid and thymol was 14 mm vs. 23 and 27 mm; 12 mm vs. 25 and 24 mm; 15 mm vs. 26 and 24 mm; 17 mm vs.25 and 32 mm; 15 mm vs.26 and 27 mm; 15 mm vs. 26 and 26 mm; 15 mm vs. 25 and 25 mm for *Escherichia coli*; *Acinetobacter baumannii*; *Pseudomonas fluorescens*; *Pseudomonas aeruginosa*; *Klebsiella pneumoniae*; *Enterobacter sakazakii* and *Enterobacter cloacae*, respectively. Except in case of combination between gallic acid and novobiocin (NV) against *Enterobacter cloacae*_15,_ the result still in resistance zone. On the other hand, chitosan had the next-highest decrease in resistance of all studied isolates. It converted susceptibility of bacterial isolates with cefotaxime (CTX), aztreonam (ATM), erythromycin (E) and azithromycin (AZM) from 100% resistant to become moderate resistant or sensitive. Meanwhile, it had no effect on PRL against *Enterobacter sakazakii*_14,_ CFP against *Escherichia coli*_1,_ *Pseudomonas aeruginosa*_10_, *Enterobacter sakazakii*_14,_ CN against *Enterobacter cloacae*_15_, SXT with *Escherichia coli*_1_, *Enterobacter sakazakii*_14_, F with *Acinetobacter baumannii*_6_ and NV with *Pseudomonas aeruginosa*_10_, *Klebsiella pneumoniae*_12_, *Enterobacter sakazakii*_14_. *Enterobacter sakazakii* was recorded the most resistant species against the studied effects of chitosan on PRL, CFP, SXT, and NV.

In contrast, EDTA showed wide variation in changing the susceptibility of different antibiotics. It was efficient in increasing inhibition zone of the completely resistant CTX, E and F toward susceptibility. While, it had a negligible effect on PRL against *Acinetobacter baumannii*_5_, *Pseudomonas aeruginosa*_10,_CFP with *Enterobacter sakazakii*_14_,ATM against *Acinetobacter baumannii*_5_, *Acinetobacter baumannii*_6_, *Enterobacter cloacae*_15_, CN with *Enterobacter cloacae*_15_, AZM against *Pseudomonas fluorescens*_7_, SXT with *Escherichia coli*_1,_ *Escherichia coli*_3_, *Acinetobacter baumannii*_6_, *Pseudomonas fluorescens*_7_, *Pseudomonas aeruginosa*_11_, NV against *Escherichia coli*_3_, *Acinetobacter baumannii*_6_, *Pseudomonas aeruginosa*_11_, *Klebsiella pneumoniae*_12_, *Enterobacter cloacae*_15_. EDTA had a negligible effect on most of the studied antibiotics against *Acinetobacter baumannii*, *Pseudomonas aeruginosa*, and *E. coli*. Finally, sorbic acid and ellagic acid had slight to moderate enhanced effect in changed the susceptibility of different antibiotics against highly resistant bacterial isolates.

In contrast, epigallocatechin gallate (ECG) alone had no effect on the activity of both cefoperazone and piperacillin against the five beta lactamase-producing bacterial isolates (Table 3), whereas quercetin was a more potent natural beta- lactamase inhibitor that decreased the resistance of originally resistant pathogenic isolates (100% resistance) to 20% resistance in the case of CFP and 80% resistance with PRL. The combination of thymol and gallic acid with natural beta lactamase inhibitors (ECG and quercetin) was effective in changing the resistance of beta lactam antibiotics (100% resistance) towards sensitivity against selected bacterial isolates. Only in the case of one of the studied *Pseudomonas aeruginosa* isolates (20%), the gallic acid with ECG had no effect on the activity of both cefoperazone and piperacillin. In addition, association of thymol or gallic acid as permeabilizers with commercial discs of piperacillin tazobactum (TZP) and cefoperazone sulbactum (SCF) exhibited high efficiency in abolishing the original resistance phenomena of the studied beta- lactamase producers.

**Table 3:** Evaluation of synergistic interaction between some natural beta lactamase inhibitors in association with selected permeabilizers with no antibacterial activity on susceptibility of beta lactamase producer isolates to different beta lactam antibiotics

Mean inhibition zone (mm) / Susceptibility break points											
Permeabilizers	Ab- alone	ECG	ECG + GA	ECG + T	Quercetin	Q + GA	Q + T	Permeabilizers	Ab-alone	Gallic acid	Thymol
^a^Antibiotics (Ab)								^b^Antibiotics (Ab)			
*Escherichia coli*_2_											
PRL ≥21	6/R	6/R	20/M	23/S	15/R	19/M	24/S	TZP ≥21	14/R	20/M	26/S
CFP ≥21	6/R	6/R	16/M	21/S	16/M	21/S	25/S	SCF ≥22	14/R	19/M	20/M
*Acinetobacter baumannii*_4_											
PRL	6/R	6/R	19/M	21/S	16/R	24/S	21/S	TZP	15/R	20/M	21/S
CFP	6/R	6/R	23/S	20/M	16/M	20/M	22/S	SCF	14/R	22/S	23/S
*Pseudomonas aeruginosa*_8_											
PRL	12/R	12/R	16/R	25/S	18/S	22/S	20/S	TZP	17/R	23/S	26/S
CFP	10/R	10/R	15/R	21/S	17/M	20/M	22/S	SCF	15/R	25/S	21/M
*Pseudomonas aeruginosa*_9_											
PRL	6/R	6/R	18/S	19/S	12/R	19/S	21/S	TZP	11/R	22/S	22/S
CFP	6/R	6/R	22/S	25/S	15/R	22/S	24/S	SCF	12/R	22/S	25/S
*Enterobacter cloacae*_13_											
PRL	6/R	6/R	20/M	23/S	15/R	20/M	24/S	TZP	20/M	30/S	27/S
CFP	6/R	6/R	18/M	21/S	18/M	20/M	23/S	SCF	18/M	25/S	23/S

*ECG* Epigallocatechin gallate, *Q* Quercetin, *GA* Gallic acid, *T* Thymol

^a^Standard discs of single β-lactam antibiotics PRL (100 μg) = Piperacillin CFP (75 μg) = Cefoperazone

^b^ Standard discs of antibiotics combined with β-lactamase inhibitors

TZP (100/10 μg) = Piperacillin/ tazobactam SCF (75/30 μg) = Cefoperazone/ sulbactam

### Effects of in vitro gamma irradiation on the studied multidrug-resistant isolates

ESBL production by all isolates was examined by the methods describe above, while only five multidrug resistant isolates were screened for the production of ESBLs by DDST and MDDT after in vitro gamma irradiation (Table 4).

An expanded zone (ghost zone) with variable increases in the diameter of the inhibition zones with respect to the effects of gamma irradiation was detected with several selected antibiotics, including amoxicillin/clavulanic acid (AMC) in DDST and piperacillin/tazobactam (TZP) in MDDT, which suggests the presence of ESBLs before and after exposure to 24.4Gy in vitro gamma irradiation for the five multidrug resistant strains, as shown in (Table 4).

Correlations between MICs obtained for cefotaxime (CTX), cefoperazone (CFP), and erythromycin (E) alone vs. after treatment with gallic acid and thymol before and after in vitro gamma irradiation demonstrate interference with the activity of these antibiotics after treatment of bacterial isolates with permeabilizers for selected non-beta lactamase producers (Table 5). Thymol showed stronger results compared to gallic acid by improving the permeability of the cell to antibiotics, along with decreases of the MIC values. Pre- and post-treatment *Escherichia coli*_*3*_, *Acinetobacter baumannii*_*5*_ and *Pseudomonas aeruginosa*_*10*_ showed changes in their MIC values with cefoperazone and erythromycin (double their values) after exposure to gamma irradiation (Table 5). From (Tables 6 and 7), in contrast to ECG, quercetin alone potentiated the activity of both cefoperazone and piperacillin 2–4 times against selected beta- lactamase producing bacterial isolates before and after exposure to in vitro gamma irradiation. On the other hand, the association between permeabilizers (gallic acid and thymol) and quercetin exhibited a prominent reducing feature (4–64 turns) compared to ECG (8–32 turns) for the MICs of both antibiotics against the selected irradiated and non-irradiated bacterial isolates.

Regarding the permeability assay, in comparing to lysozyme itself, a marked lysis-promoting effect was observed in *Pseudomonas aeruginosa*_10_ and *Escherichia coli*_3_ in the presence of gallic acid and thymol for Triton X-100 (1%) and both concentrations of SDS tested. Overall, the effects of gallic acid as a sensitizing agent were more pronounced. Permeability of the outer membrane of the tested non-beta lactamase producers was not affected (statistically nonsignificant differences) by radiation (Table 8)

**Table 4:** Effects of gamma irradiation on ESBL production by five ESBLs-producing bacterial strains

Isolate No./ Species of microorganism	Radiation	Half of inhibition zone (mm) in absence AMC / adjacent to AMC disk (DDST) Mean ± SD (SE)					Half of inhibition zone (mm) in absence TZP/adjacent to TZP disk (MDDT) Mean ± SD (SE)		
		AMC	CTX	CAZ	FEP	ATM	TZP	CAZ	FEP
2/ *Escherichia coli*	Before	5.5 ± 0.5(0.3)	3 ± 0.5(0.3) /7 ± 0.5(0.3)	3 ± 0.3 (0.2) /8 ± 0.6 (0.3)	3 ± 0.2 (0.1) /3 ± 0.6 (0.3)	3 ± 0.8 (0.5) /3 ± 0.1 (0.1)	7 ± 0.8 (0.5)	3 ± 0.2 (0.1) /9 ± 0.2 (0.1)	3 ± 0.5(0.3) /6 ± 0.5(0.3)
	After	6 ± 0.5(0.3)	3 ± 0.2 (0.1) /7 ± 0.2 (0.1)	3 ± 0.5(0.3) /8 ± 0.7 (0.4)	5 ± 0.4 (0.2) /5 ± 0.1 (0.1)	3 ± 0.5(0.3) /3 ± 0.7 (0.4)	9 ± 0.2 (0.1)	3 ± 0.4 (0.2) /9 ± 0.2 (0.1)	5 ± 0.3 (0.2) /8 ± 0.2 (0.1)
	% of change	8.3	0.0 /0.0	0.0 /0.0	40 /40	0.0 /0.0	22.2	0.0 /0.0	40 /25
	(*p*-value)	0.288	1.000 /1.000	1.000 /1.000	<0.001** /0.005*	1.000 /1.000	0.014*	1.000 /1.000	0.004* /0.003*
4/ *Acinetobacter baumannii*	Before	5 ± 0.4 (0.2)	3 ± 0.5(0.3) /3 ± 0.4 (0.2)	3 ± 0.2 (0.1) /3 ± 0.4 (0.2)	3.5 ± 0.5(0.3) /10 ± 0.6 (0.3)	3 ± 0.3 (0.2) /7 ± 0.7 (0.4)	7.5 ± 0.5(0.3)	3 ± 0.7 (0.4) /10 ± 0.2 (0.1)	3.5 ± 0.4 (0.2) /3.5 ± 0.5(0.3)
	After	5 ± 0.6 (0.3)	5 ± 0.3 (0.2) /6 ± 0.6 (0.3)	5 ± 0.4 (0.2) /6 ± 0.2 (0.1)	4 ± 0.5(0.3) /11 ± 0.8 (0.5)	3 ± 0.8 (0.5) /7 ± 0.2 (0.1)	9.5 ± 0.5(0.3)	5 ± 0.5(0.3) /13 ± 0.4 (0.2)	4 ± 0.2 (0.1) /5.5 ± 0.3 (0.2)
	% of change	0.0	40 /50	40 /50	12.5 /9.1	0.0 /0.0	21.1	40 /23.1	12.5 /36.4
	(*p*-value)	1.000	0.004*/ 0.002*	<0.001** / <0.001**	0.288 /0.158	1.000 /1.000	0.008*	0.016* / <0.001**	0.125 /0.004*
8/ *Pseudomonas aeruginosa*	Before	3 ± 0.9 (0.5)	3 ± 0.2 (0.1)/3 ± 0.5(0.3)	4.5 ± 0.4 (0.2) /10 ± 0.2 (0.1)	8 ± 0.4 (0.2) /12 ± 0.2 (0.1)	6 ± 0.3 (0.2) /6 ± 0.5(0.3)	8.5 ± 0.5(0.3)	4.5 ± 0.5(0.3) /11 ± 0.2 (0.1)	8 ± 0.2 (0.1) /12 ± 0.4 (0.2)
	After	5 ± 0.3 (0.2)	5 ± 0.4 (0.2)/6 ± 0.2 (0.1)	6.5 ± 0.2 (0.1)/13 ± 0.5(0.3)	9 ± 0.2 (0.1) /13.5 ± 0.5(0.3)	7 ± 0.4 (0.2)/8 ± 0.2 (0.1)	12 ± 0.3 (0.2)	6 ± 0.2 (0.1) /13.5 ± 0.5(0.3)	9 ± 0.1 (0.1) /14 ± 0.2 (0.1)
	% of change	40	40 /50	30.8 /23.1	11.1 /11.1	14.3 /25	29.2	25 /18.5	11.1 /14.3
	(*p*-value)	0.22*	<0.001** / <0.001**	<0.001** / <0.001**	0.018* /0.008*	0.026* /0.003*	<0.001**	0.008* / <0.001**	<0.001** / <0.001**
9/ *Pseudomonas aeruginosa*	Before	3 ± 0.3 (0.2)	3 ± 0.5(0.3) /3 ± 0.2 (0.1)	3 ± 0.4 (0.2) /9 ± 0.2 (0.1)	3 ± 0.2 (0.1) /8 ± 0.3 (0.2)	8 ± 0.2 (0.1) /8 ± 0.5(0.3)	5.5 ± 0.5(0.3)	3 ± 0.2 (0.1) /8 ± 0.4 (0.2)	3 ± 0.2 (0.1) /7 ± 0.1 (0.1)
	After	4.5 ± 0.5(0.3)	4.5 ± 0.5(0.3) /5 ± 0.2 (0.1)	5 ± 0.2 (0.1) /11 ± 0.4 (0.2)	5 ± 0.5(0.3) /11 ± 0.2 (0.1)	9 ± 0.2 (0.1) /10 ± 0.3 (0.2)	6.5 ± 0.3 (0.2)	5 ± 0.1 (0.1) /10 ± 0.2 (0.1)	5 ± 0.5(0.3) /10 ± 0.1 (0.1)
	% of change	33.3	33.3 /40	40 /18.2	40 /22.3	11.1 /20	15.4	40 /20	40 /30
	(*p*-value)	0.011*	0.021* / <0.001**	<0.001** / <0.001**	0.003* / <0.001**	0.004* /0.004*	0.041*	<0.001** / <0.001**	0.003* / <0.001**
13/ *Enterobacter cloacae*	Before	4 ± 0.1 (0.1)	3 ± 0.2 (0.1) /3 ± 0.5(0.3)	7 ± 0.1 (0.1) /11 ± 0.4 (0.2)	6 ± 0.2 (0.1) /11 ± 0.4 (0.2)	6 ± 0.2 (0.1) /6 ± 0.1 (0.1)	10 ± 0.2 (0.1)	7 ± 0.1 (0.1) /12 ± 0.2 (0.1)	6 ± 0.1 (0.1) /10 ± 0.2 (0.1)
	After	4 ± 0.5(0.3)	4 ± 0.1 (0.1) /4 ± 0.3 (0.2)	8.5 ± 0.4 (0.2)/13 ± 0.2 (0.1)	6.5 ± 0.1 (0.1) /12 ± 0.1 (0.1)	6 ± 0.2 (0.1) /7 ± 0.3 (0.2)	12 ± 0.3 (0.2)	8.5 ± 0.1 (0.1) /14 ± 0.2 (0.1)	6.5 ± 0.2 (0.1)/10.5 ± 0.3 (0.2)
	% of change	0.0	25 /25	17.6 /15.4	7.5 /8.3	0.0 /14.3	16.7	17.6 /14.3	7.5 /4.8
	(*p*-value)	1.000	<0.001** / 0.041*	0.003* / <0.001**	0.018* /0.014*	1.000 /0.005*	<0.001**	<0.001** / <0.001**	0.018* /0.074

*DDST* Double disk synergy test, *MDDT* Modified double disk synergy test *p*-value significant <0.05

AMC (20/10 μg) = Amoxicillin /clavulanic Acid CTX (30 μg) = Cefotaxime *p*-value non significant > 0.05

CAZ (30 μg) = Ceftazidime FEP (30 μg) = Cefepime *p*-value highly significant <0.001**

ATM (30 μg) = Aztreonam TZP (100/10 μg) = Piperacillin / tazobactam

**Table 5:** Effect of in vitro gamma irradiation on MIC of different antibiotics alone or combined with permeabilizers of non-beta lactamase producer bacterial isolates

Antibiotics	MICs (mg/L) before irradiation			MICs (mg/L) after irradiation		
Bacterial species	Ab-Alone	+Gallic acid	+Thymol	Alone	+Gallic acid	+Thymol
*Escherichia coli*_*3*_						
CTX	64	8	4	64	8	4
CFP	64	16	16	128	32	32
E	128	32	32	256	64	64
*Acinetobacter baumannii*_*5*_						
CTX	32	8	8	32	8	8
CFP	32	8	8	64	16	16
E	64	32	16	128	64	32
*Pseudomonas aeruginosa*_*10*_						
CTX	128	32	32	128	32	32
CFP	128	32	32	256	64	64
E	128	128	64	256	256	128
*Enterobacter sakazakii*_*14*_						
CTX	8	2	4	8	2	4
CFP	32	16	8	32	16	8
E	64	16	16	64	16	16

*CTX* Cefotaxime, *CFP* Cefoperazone, *E* Erythromycin

**Table 6:** Evaluation of MICs of certain antibiotics alone and with beta lactamase inhibitors alone (ECG) or in combination with gallic acid or thymol and selected permeabilizers before and after in vitro gamma irradiation

Antibiotic (Ab)	MICs (mg/L) before irradiation					MICs (mg/L) after irradiation				
	Ab-alone	+ECG	+ECG+ Gallic acid	+ECG +Thymol	antibiotic disc with beta-lactamase inhibitors	Ab- Alone	+ECG	+ECG+ Gallic acid	+ECG + Thymol	antibiotic disc with beta-lactamase inhibitors
Bacterial beta lactamase producer										
*Escherichia coli*_*2*_										
CFP	64	64	8	4	^a^32	64	64	8	4	^a^32
PRL	128	128	16	4	^b^16	128	128	16	4	^b^16
*Pseudomonas aeruginosa*_*8*_										
CFP	128	128	16	8	64	128	128	16	8	64
PRL	128	128	8	8	64	128	128	8	8	64
*Enterobacter cloacae*_13_										
CFP	32	32	4	4	16	64	64	8	8	32
PRL	64	64	4	4	0.5	128	128	8	8	1

*Ab* Antibiotic, *ECG* Epigallocatechin gallate, *CFP* Cefoperazone

PRL = Piperacillin. ^**a**^ SCF = Cefoperazone/ sulbactum. ^b^ TZP = Piperacillin/ tazobactam

**Table 7:** Evaluation of MICs of certain antibiotics alone and in the presence of beta lactamase inhibitors alone (quercetin) or in combination with gallic acid or thymol and selected permeabilizers before and after in vitro gamma irradiation

Antibiotics (Ab)	MICs (mg/L) before irradiation					MICs (mg/L) after irradiation				
	Ab-alone	+Q	+Q + Gallic acid	+Q + Thymol	Antibiotic disc with beta-lactamase inhibitors	Ab-alone	+Q	+Q + Gallic acid	+Q + Thymol	antibiotic disc with beta-lactamase inhibitors
Bacterial beta lactamase producer										
*Escherichia coli*_*2*_										
CFP	64	32	8	2	^a^32	64	32	8	2	32
PRL	128	32	8	4	^b^16	128	32	8	4	16
*Pseudomonas aeruginosa*_*8*_										
CFP	128	32	8	2	64	128	32	8	2	64
PRL	128	64	8	8	64	128	64	8	8	64
*Enterobacter cloacae*_13_										
CFP	32	16	8	4	16	64	64	32	8	32
PRL	64	32	2	1	0.5	128	64	4	2	1

*Q* Quercetin, *CFP* Cefoperazone, *PRL* Piperacillin

^a^SCF = Cefoperazone/ sulbactum ^b^ TZP = Piperacillin/ tazobactam

**Table 8:** Effects of different lytic agents on the outer membrane permeability of *Pseudomonas aeruginosa*_10_ / *Escherichia coli*_3_ pretreated with permeabilizers and gamma irradiation

Lytic agent	Conc.	Relative turbidity (%) *P. aeruginosa*_10_ / *E. coli*_3_			
		Control-	Gallic acid	Thymol	Radiation
Lysozyme	10 μg/mL	100 ± 1 /101 ± 1	99 ± 1/ 92 ± 1	100 ± 1/ 98 ± 0.6	100 ± 0.6/ 101 ± 2
Triton X-100	0.1%	100 ± 2 /103 ± 3	93 ± 3/86 ± 3	96 ± 1/ 90 ± 3	100 ± 1/ 101 ± 1.2
Triton X-100	1%	99 ± 3 /105 ± 0.6	81 ± 1/77 ± 2	84 ± 3/ 74 ± 4	99 ± 2/ 103 ± 3
SDS	0.1%	92 ± 1 /91 ± 2	58 ± 2/ 60 ± 1	60 ± 3/ 59 ± 3	92 ± 1/ 91 ± 5
SDS	1%	78 ± 0.6 / 80 ± 2	26 ± 1/ 42 ± 2	28 ± 2/ 50 ± 0.6	79 ± 2/ 82 ± 2

The value of control cells without lytic agents was set at 100%

.

**Fig. 1:**
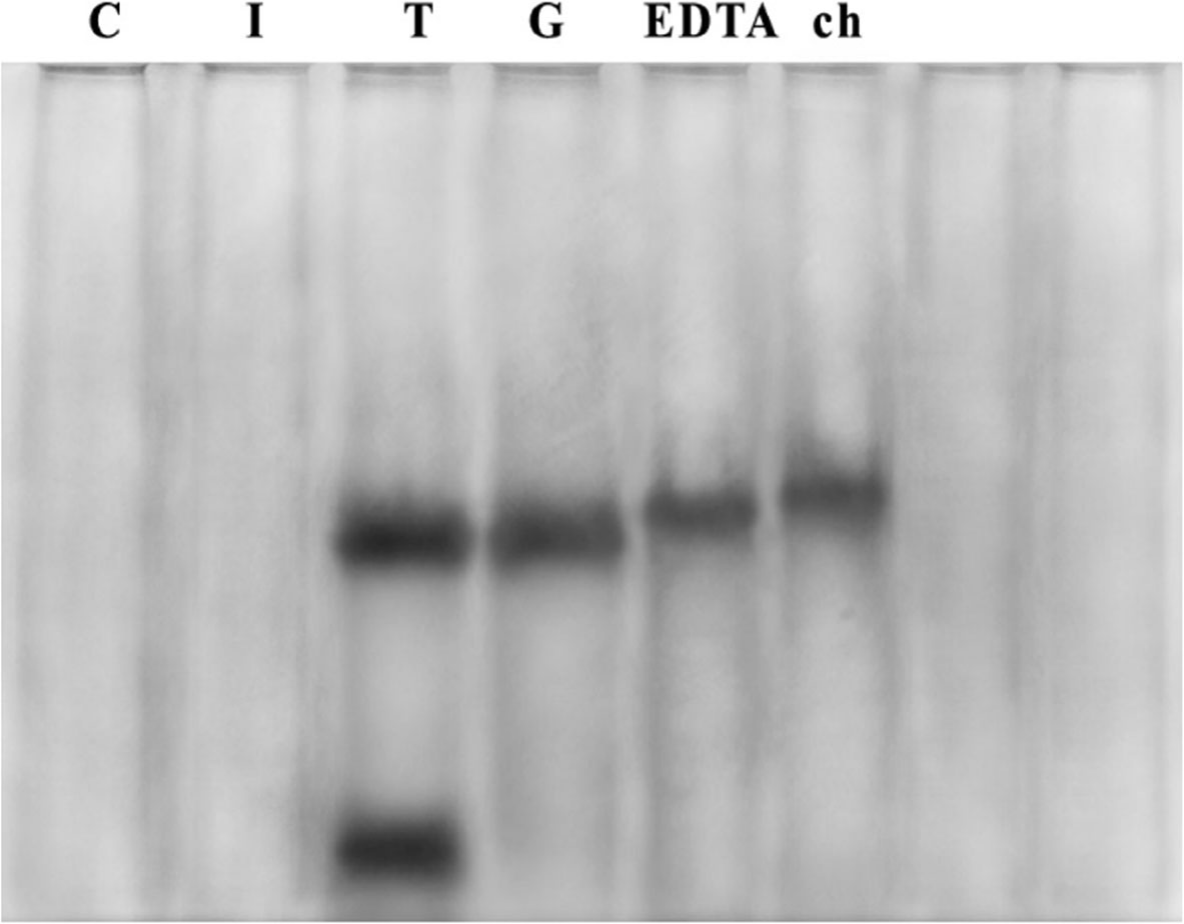
SDS-PAGE of Proteinase K-treated cell-free supernatants of *Escherichia coli*_3_ (silver staining). Lanes: 1, untreated (control); 2, irradiated supernatant; 3, thymol supernatant; 4, gallic acid supernatant; 5, EDTA supernatant; 6, chitosan supernatant

Proteinase K-treated samples of cell-free supernatants derived from suspensions of *Escherichia coli*_3_ treated with permeabilizers or with radiation were electrophoresed. From the results shown in Fig. 1, no band was detected in the supernatants of control or irradiated samples. In contrast to the supernatants of untreated cells, the thymol-treated cells released a considerable amount of LPS into the supernatant. There was one prominent band present in the supernatant of samples treated with gallic acid, EDTA and chitosan. On the basis of visual estimation of the intensity of staining, the supernatants from thymol- and gallic acid-treated bacteria contained more LPS than those derived from treatments with chitosan and EDTA.

From (Table 9), it is clear that cefoperazone treatment did not alter the OM permeability when used alone. In contrast, the combination of cefoperazone and thymol increased the OM permeability of two beta lactamase producers to antibiotics. The differences in absorbance between control and posttreatment samples of these combinations with thymol were highly significant in *Pseudomonas aeruginosa*_8_ (*P* < 0.001) and in *Escherichia coli*_2_ (*P* < 0.002). These results indicate that increased perplasmic antibiotic concentrations that were inactivated by beta lactamase enzyme. There was no marked difference between controls (without any treatment) and the combination of cefoperazone and thymol in the presence of quercetin, indicating that these compounds showed marked beta lactamase inhibitory activity. Low-dose gamma radiation had highly significant effects (*P* < 0.001) on the tested species via all decolorization methods.

**Table 9:** Beta lactamase assays in the presence of permeabilizer (thymol) and natural beta lactamase inhibitor (quercetin) for irradiated and non-irradiated *Pseudomonas aeruginosa*_8_ / *Escherichia coli*_2_

Conc.	control	*Pseudomonas aeruginosa*_8_ / *Escherichia coli*_2_					
		^a^Cefoperazone		^a^Cefoperazone + thymol		^a^Cefoperazone +thymol+quercetin	
		Before	After	Before	After	Before	After
128/64	0.271/0.273	0.123 ± 0.001/ 0.148 ± 0.001	0.142 ± 0.001/ 0.161 ± 0.002	0.037 ± 0.002/ 0.049 ± 0.006	0.044 ± 0.0006/ 0.053 ± 0.002	0.196 ± 0.002/ 0.215 ± 0.001	0.211 ± 0.0006/ 0.221 ± 0.001
64 /32	0.263 /0.263	0.119 ± 0.0006/ 0.139 ± 0.002	0.133 ± 0.003/ 0.160 ± 0.001	0.030 ± 0.001/ 0.048 ± 0.001	0.040 ± 0.001/ 0.050 ± 0.0005	0.183 ± 0.003/ 0.206 ± 0.001	0.203 ± 0.001/ 0.213 ± 0.0006
32/16	0.256/0.258	0.115 ± 0.001/ 0.128 ± 0.0006	0.130 ± 0.0006/ 0.148 ± 0.002	0.02 ± 0.001/ 0.045 ± 0.001	0.039 ± 0.0006/ 0.048 ± 0.002	0.179 ± 0.001/ 0.197 ± 0.001	0.20 ± 0.006/ 0.203 ± 0.001

Control = Cefoperazone antibiotic in a solution of B-CHR-TMB

^a^ Mean results of triplicate experiments

## Discussion

Nosocomial infections are primarily caused by Gram-negative bacteria. Due to their intrinsic and acquired capabilities to develop resistance to antimicrobial agents, they are difficult to treat [24]. The emergence and spread of multidrug resistance (MDR) among Gram-negative bacilli, including Enterobacteriaceae, *Pseudomonas* and *Acinetobacter* species, could be explained by [25] who found that higher levels antibiotic resistance are generally not attributable to intrinsic bacterial resistance alone; rather, it is attributable to a synergistic relationship between both the impermeability of the outer membrane and other extrinsic resistance factors, such as the enzymatic inactivation of antibiotics. Gram-negative isolates producing ESBL confer resistance to all β-lactam agents and to several non β-lactam-based antibiotics, such as aminoglycosides, flouroquinolones and sulfamethoxazole-trimethoprim, because most plasmids not only contain DNA encoding ESBL enzymes but also carry a gene that confers resistance to several non β-lactams antibiotics. This makes it exceedingly difficult to treat the infections they produce**.** Therefore, detection of ESBL-producing strains is important because their spread within hospitals may lead to endemic occurrence and repeated outbreaks, in addition to limiting therapeutic options. From this point of view, this study was performed to detect from 15 isolates belonging to 5 genera and 7 species ESBL-producers using several methods. The obtained results revealed that the four screening methods used were adequate detection methods, with agreement reached 100% for Nitrocefin testing vs. DDST, MDDT, Comb-CFP-SCF and for Comb- PRL-TZP.

As a result of gamma irradiation, the ability of the tested isolates to produce ESBL was altered after exposure to in vitro gamma irradiation **(**Table 4**)** in DDST, MDDT and beta lactamase assays, which was in accordance with [26]. The effects of radiation on ESBL production are significant and may be because ESBL production is encoded by genes that are predominantly located on plasmids. Furthermore, radiation may also cause a number of lethal and sublethal effects in the other structures of cells, such as enzymes and plasmids**.** Outer membrane permeability and β-lactamase are key factors mediating the resistance of bacteria to antibiotics. Rapid penetration of antibiotics is an important factor affecting bactericidal activity, and effective permeabilization of the outer membrane may overcome intrinsic resistance pathways. Permeabilizers themselves may not be inherently bactericidal, but they may potentiate the activity of other compounds, thus acting synergistically [20]. Therefore, the current study sought to overcome the challenge of antibiotic resistance by using a combination of non-antibacterial concentrations of natural products (phytochemicals) and certain antibiotics to increase their efficacy against pathogenic Gram-negative bacilli. These combinations could reduce the toxicity of the drugs used, avoiding the emergence of resistant variants that might otherwise arise during treatment, and may have synergistic effects for combating infections caused by Gram-negative bacteria [27]. The results obtained in this study demonstrate that the studied natural products (permeabilizers) increased the susceptibility of the target strains to different antibiotics (including hydrophobic antibiotics, such as erythromycin, azithromycin, sulphamethoxazole trimethoprime, nitrofurantoin and novobiocin). These results may indicate that the outer membrane barrier was disturbed by the permeabilizers, which significantly enhanced the effects of the antibiotics. In addition, effective OM permeabilizers sensitize Gram-negative bacteria to hydrophobic antibiotics, including erythromycin and novobiocin, which are generally not useful in treating Gram-negative bacterial infections as they traverse the OM ineffectively. The phytochemicals tested (gallic acid and thymol) had clear synergistic activities with different classes of antibiotics, resulting in increased activity and reduced minimum effective doses of antibiotics against Gram-negative bacterial species. This is consistent with results obtained by [28] Gallic acid has proven to be an efficient permeabilizer. The OM-disintegrating activity of gallic acid has been suggested to be based on the chelation of divalent cations and the partial hydrophobicity of this product, which promotes membrane destabilization. Additionally, this compound has been reported to cause irreversible changes in membrane properties through hydrophobicity changes, decreases in negative surface charges, and the occurrence of local ruptures or pore formation in the cell membranes [3]. Thymol can be effective against microorganisms through its lipophilic action on the cellular membrane, causing the dispersion of the polypeptide chains of the cellular membrane and destabilizing the permeability of the cell membrane. Thymol has prominent OM-disintegrating properties, as indicated by its enhancing effect on LPS release, but does not affect the chelation of cations. Thymol integrates within the polar head groups of the lipid bilayer, inducing alterations of the cell membrane. At low thymol levels, the membrane can adapt its lipid profile to maintain membrane function and structure [20].

In the MIC and beta lactamase assays, the combination of permeabilizers (thymol and gallic acid) with antibiotics and beta lactamase inhibitors (quercetin) showed high permeability rates, low MIC values and higher overall susceptibility of the tested bacteria compared to treatment with antibiotics alone or antibiotics in combination with natural beta lactamase inhibitors (Table 7). This finding is consistent with [29], who reported that the efficacy of a beta lactamase inhibitor/beta lactam combination depends on many parameters, such as the intrinsic activities of both components against their respective target enzymes. The activity of beta lactamase inhibitors and their penetration rates across the outer membrane are also considered important factors that determine how effectively they neutralize periplasmic beta-lactamase. However, efficient penetration of the inhibitor through the outer membrane is essential to fully realize its inhibitory potency and thus maximize the antibacterial activity of the partner antibiotic. In this study, the obtained results **(**Table 7**)** provide a unique example that quercetin, which has no beta lactam structure, can reverse bacterial resistance to beta lactams more effectively than traditional beta lactamase inhibitors, such as clavulanic acid or sulbactum, as quercetin (beta lactamase inhibitor) reduced MICs of studied antibiotics (i.e., cefoperazone and piperacillin) against the tested ESBL-producers (*E. coli*, *Pseudomonas aeruginosa* and *Enterobacter cloacae*), which represent several of the most dangerous and problematic MDR bacterial pathogens; these findings are in accordance with [30]. This could be because of the structural dissimilarity between quercetin and β-lactam antibiotics; this compound is therefore unlikely to induce β-lactamase production, while clavulanate and sulbactum share the same key structure with beta lactam antibiotics and may cause considerable induction of beta lactamase expression; indeed, an increase in their concentration was followed by an elevation in beta lactamase production. This means that the currently available beta lactamase inhibitors can also lose their activity by the same mechanism as the beta lactam antibiotics [31].

Similarly, epigallocatechingallate (EGCG) had no intrinsic effect on the studied Gram-negative bacteria producing ESBLs; however, in combination with permeabilizers that facilitate its entry across the Gram–negative bacterial outer membrane, it acted as a potent inhibitor **(**Table 6**)**. This may be explained by the differences in combinational effects, which have been confirmed to be related to the cellular locations of the enzymes. The low catechin susceptibility of Gram-negative bacteria may be at least partially attributable to the presence of lipopolysaccharide acting as a barrier [32].

Additionally, differences in MICs of the tested isolates in relation to gamma irradiation were strain-dependent, with *Enterobacter cloacae*_13_ proving more susceptible to gamma radiation compared with the rest of the tested beta lactamase-producing microorganisms **(**Tables 6 and 7**)**. The relative sensitivity or resistance of different microorganisms to ionizing radiation is based on their respective D values. A D10 value is defined as the radiation dose required to reduce the population by 10-fold (one log cycle) or the dose required to kill 90% of the total viable number [33].

The presence and features of lipopolysaccharide (LPS) molecules in the outer leaflet of the membrane result in Gram-negative bacteria having an inherent resistance to hydrophobic antibiotics (e.g., macrolides and novobiocins) and detergents (e.g., bile salts, SDS and Triton X-100). Sensitization of Gram-negative cells to cell lysis induced by detergents (e.g., sodium dodecyl sulfate [SDS] and Triton X-100) as well as lysis by lysozyme and deoxycholate, are indications of weakening of the OM [21]. Therefore, the activity of thymol and gallic acid as membrane permeabilizers were confirmed in this study by permeability assay. Both permeabilizers were strongly able to increase the permeability of the outer membrane to lytic agents in the tested bacterial isolates (*P* < 0.001) (Table 8), which is in accordance with previous findings of [25].

Purified LPS was characterized by SDS-PAGE electrophoresis followed by silver staining. The results of silver staining clearly showed a ladder pattern of bands with multiple rungs, which is characteristic of the smooth type of Gram-negative bacteria due to the carbohydrate chain length variation of the O-antigen segment. Sliver staining is a highly sensitive method capable of detecting as little as 1 ng LPS and is routinely used for visualization of the band pattern of purified LPS [34]. The results presented in this study confirm that, in comparison to chitosan and EDTA, thymol and gallic acid are incredibly potent OM-disintegrating agents, as evidenced by their ability to cause LPS release. This could be explained by the phenolic character of thymol; phenols are known for their membrane disturbing activities, their reversible permeabilizing effect of chitosan, and the high concentrations of EDTA (1 mM) required to be strongly active in exerting its OM- disintegrating action [35].

The efficacy of β-lactam antibiotics against Gram-negative bacteria has been hypothesized to depend on their rate of penetration across the outer membrane and their degree of resistance to β-lactamase inactivation. A very slow penetration through the outer membrane would not be sufficient to build up an effective periplasmic concentration**.** A possible explanation for the decrease in absorbance of cefoperazone antibiotic in TMB solution **(**Table 9**)** in the presence of the selected beta lactamase producers may be due to the presence of extracellular enzyme, which was initially thought to stem exclusively from lysed and broken cells. Such extracellular activity may contribute to inactivation of the beta-lactam antibiotic by opening the beta lactam ring [36]**.** Exposure of bacterial cells to ionizing radiation presents an additional stress to the cells that tends to disturb their organization. Nucleic acids, especially DNA, are the primary target for cell damage induction by ionizing radiation. Gamma radiation induces three types of damage in DNA: single-strand breaks, double-strand breaks and nucleotide damage, which include base damage and damage in the sugar moiety. Base damage is a major component of damage induced by ionizing radiation. Gamma irradiation also affects protein fingerprinting and enzymes. As a result of radiation, the plasmid DNA was partially damaged; at the same time, the radiation may activate the expression of other genes, including certain genes encoding antibiotic resistance, which was reflected by the increase of relevant resistance compared to non-irradiated samples [37].

## Conclusions

This study suggests that thymol and gallic acid are potent OM-disintegrating agents, as evidenced by their ability to release LPS and sensitize Gram-negative bacilli to different lytic agents. Several natural compounds have potential activity as beta lactamase inhibitors (quercetin) and may lead to the development of more potent beta lactamase inhibitors for new antimicrobial combinations. Determination of MICs for three selected antibiotics with all tested strains alone (cefotaxime, cefoperazone, erythromycin) before and after irradiation showed an increase in the MIC of cefoperazone and erythromycin after irradiation to double its value with *Escherichia coli*_*3*_, *Acinetobacter baumannii*_*5*_ and *Pseudomonas aeruginosa*_*10*_*.* The results obviously showed that, the combination of selected antibiotics with the most potent permeabilizers (gallic acid and thymol) decreased MIC values, indicating weaken the OM. Exposure of cancer patients to radiotherapy in cancer treatment regimens affects the pathogenicity of microorganisms as well as their susceptibility to different antimicrobial agents. Therefore, physicians and medical stuff should closely heed the results of microbiological laboratory testing regarding microbial infection and antimicrobial susceptibility when providing radiotherapy treatment.

## Data Availability

Not applicable.

## Abbreviations

AK: Amikacin
AMC: Amoxicillin/clavulanicacid
ATM: Aztreonam
AZM: Azithromycin
BSI: Blood stream infection
CFP: Cefoperazone
CT: Colistin sulfate
CTX: Cefotaxime
E: Erythromycin
EA: Ellagic acid
EGCG: Epigallocatechin-3-O-gallate
ESBLs: Extended spectrum β-lactamase
F: Nitrfurantoin
FEP: Cefepime
FOX: Cefoxitin
GA: Gallic acid
GN: Gentamycin
IPM: Imipenem
LPS: Lipopolysaccharide
MDR: Multi-drug resistant
MEM: Meropenem
MIC: Minimum inhibitory concentration
MR: Moderate resistant
mRNA: Messenger ribonucleic acid
NA: Nalidixic acid
NV: Novobiocin
OM: Outer membrane (OM)
PRL: Piperacillin
SAM: Amoacillin- Sulbactam
SXT: Sulphamethoxazole trimethoprime
TZP: Pipracellin-Tazobactam
